# The health-related quality of life in Iranian patients with COVID-19

**DOI:** 10.1186/s12879-021-06170-z

**Published:** 2021-05-20

**Authors:** Cyrus Alinia, Safura Yaghmaei, Farman Zahir Abdullah, Asad Ahmadi, Nasrin Samadi, Sima Pourteimour, Hossein Safari, Hassan Mahmoodi, Ghobad Moradi, Bakhtiar Piroozi

**Affiliations:** 1 Department of Health Management and Economics, School of Public Health, Urmia University of Medical Sciences, Urmia, Iran; 2 Nursing Care Research Center, Semnan University of Medical Sciences, Semnan, Iran; 3 Special Education Department, College of Education and Language, Charmo University, Chamchamal, Kurdistan Region Iraq; 4 Social Determinants of Health Research Center, Research Institute for Health Development, Kurdistan University of Medical Sciences, Sanandaj, Iran; 5 Student Research Committee, Urmia University of Medical Sciences, Urmia, Iran; 6 Department of Nursing, School of Nursing and Midwifery, Semnan University of Medical Sciences, Semnan, Iran; 7 Health Promotion Research Center, Iran University of Medical Science, Tehran, Iran

**Keywords:** COVID-19, Health-related quality of life, Utility value, Disease disutility

## Abstract

**Background:**

COVID-19 is a public health emergency with a high mortality rate and it reduces the patient’s Health-Related Quality of Life (HRQoL) significantly. This effect is measured in the current study.

**Methods:**

In a cross-sectional study in Iran, 320 randomly selected treated patients from COVID-19 were studied. To collect the required data, we applied a questionnaire that included socio-demographic factors, clinical characteristics, and questions on the patients’ HRQoL. Time trade-off (TTO) approach was used to measure the lost HRQoL attributed to COVID-19. Besides, we applied a two-limit Tobit regression model to determine the effects of the socio-demographic factors on patients’ health utility and the visual analogue scale approach was used to estimate the perceived total current health status.

**Results:**

The overall mean (SE) and median (IQR) of the health utility values were 0.863 (0.01) and 0.909 (0.21) respectively. These values for the traders (those who were willing to lose a part of their remaining time of life to avoid the disease) were estimated at 0.793 (0.01) and 0.848 (0.17), respectively. The lowest amount of utility value belonged to the elderly (mean (SE) = 0.742 (0.04); median (IQR) = 0.765 (0.42)) and those living in rural areas (mean (SE)) = 0.804 (0.03); median (IQR) = 0.877 (0.30)). The univariate analysis showed that age, place of residence, and household size had a statistically significant effect on health utility. Moreover, findings of the regression analysis indicated that the participants’ age and hospitalization status were the key determinants of COVID-19 health utility value.

**Conclusion:**

COVID-19 is associated with a substantial and measurable decrease in HRQoL. This decline in HRQoL can be directly compared with that induced by systemic health states.

**Supplementary Information:**

The online version contains supplementary material available at 10.1186/s12879-021-06170-z.

## Background

Novel coronavirus disease 2019 (COVID-19) caused by severe acute respiratory syndrome virus coronavirus 2 (SARS-CoV-2) was first identified in the world in Wuhan, China in December 2019. About 3 months later, the outbreak of the virus was declared by the World Health Organization (WHO) as a global health crisis and a pandemic [1, 2]. COVID-19 is an acute respiratory syndrome with common symptoms including fever, cough, shortness of breath, muscle aches, tiredness, sore throat, headache, and loss of smell and taste. Although, this disease is mild in most people and can be treated with no special treatment, in some people it can lead to serious illness and even death [3, 4]. The mortality rate and severity of the disease vary with age, underlying medical conditions such as cardiovascular disease, diabetes, cancer, and chronic respiratory disease and other health conditions [5–7]. Evidence suggests that COVID-19 has a negative effect on physical and mental health and health-related quality of life of COVID-19 patients [8–10].

Severe acute respiratory infections lead to illness, death, and hospitalization of millions of people worldwide annually, and are one of the main reasons for the referral and hospitalization of the elderly and children [11]. By July 8, 2020, SARS CoV had infected approximately 12 million people worldwide and killed over 540,000 [12]. The rapid spread of COVID-19 worldwide has placed a heavy burden on health systems and many countries are facing a shortage of hospital equipment and facilities, such as intensive care beds and ventilators. In addition, the number of hospitalized patients with COVID-19 has exceeded the standard capacity of hospitals [13–15]. Despite the urgent need for appropriate evidence for key decision-making, information about COVID-19 in the world is still limited. Appropriate epidemiological information is needed for proper policy-making and to control COVID-19 [16]. One of this information is the utility value of the disease. Without knowing the utility value of the disease, it is not possible to calculate the burden of the disease and conduct economic evaluation studies of preventive and therapeutic interventions [17]. Utility value for a disease indirectly indicates a patient’s Health-Related Quality of Life (HRQoL) level [18, 19]. Depending on the severity of the disease, age, and the underlying medical conditions, COVID-19 imposes various physical and mental limitations on patients [4, 10, 20].

The aim of the current study was to calculate the disease utility for different degrees of Covid-19 in different socio-economic subgroups. The findings of the study can be used in economic evaluation studies and also in calculating the burden of Covid-19 at different geographic levels.

## Methods

### Study design and participants

This cross-sectional, multicenter study was conducted on treated patients from COVID-19 at three hospitals in provinces of Kurdistan, West Azerbaijan, and Hamadan in western and northwestern Iran between May 21, 2020, and June 18, 2020. The study population included 320 randomly selected individuals who were discharged from hospitals over the past 2 weeks. To avoid overestimating the disutility value for COVID-19 and to be able to extract people’s logical and real judgments about the effects of the disease on the HRQoL, we studied the newly recovered patients. To calculate the minimum required sample size, we applied the Walters [21] formula for non-normally distributed continuous data. In this calculation, we considered a two-tailed 5% significance level, 80% power, effect size (P_Noether_) of 0.57 (consistent with those used in common association analyses), and response rate of 80% which gives the estimated number of subjects as 320.

$$ n=\frac{2{\left({Z}_{1-\raisebox{1ex}{$\alpha $}\!\left/ \!\raisebox{-1ex}{$2$}\right.}+{Z}_{1-\beta}\right)}^2}{6{\left({P}_{Noether}-0.5\right)}^2} $$

Diagnosis of COVID-19 was done by trained laboratory staff using real-time reverse transcriptase polymerase-chain-reaction (RT PCR) assay for nasal pharyngeal swab specimens based on World Health Organization interim guidance [22]. We included the laboratory-confirmed cases in the analysis and only patients who did not wish to participate in the study were excluded. For patients under the age of 18 and those who were unable to speak, the interview was conducted with the most literate member of his/her family as a proxy, who was over 15 years old.

### Data collection

To collect the required data, we applied a validated Persian language questionnaire [17, 23] that included sociodemographic factors as well as questions on the patients’ HRQoL and clinical characteristics. The first two sections were completed through a telephone interview with the patients and the third section was completed by studying the patient’s medical records. The telephone interviews were conducted by three trained nurses who were working at the hospital wards dedicated to patients with COVID-19 under supervision of the principal investigator. Sociodemographic variables included age, gender, place of residence (urban/ rural), education (illiterate/ non-university (elementary, middle, and high school)/ university), having a job (yes/ no), marital status (single/ married) during the disease, household size, standardized monthly household expenditure (monthly household expenditure is divided by square root of household size), and having basic health insurance (yes/ no). This part of the questionnaire was completed self-reportedly.

Clinical characteristics cover information on having an underlying disease (yes/ no), the patient’s hospitalization condition during the disease (quarantine at home/ hospitalized at general wards/ hospitalized at Intensive Care Unit (ICU) without intubation/ and hospitalized at ICU with intubation), hospitalization days, arterial blood hemoglobin oxygen saturation (SpO2), and level of lung involvement (non or minor/ poor/ moderate/ severe). SpO2 was measured by pulse oximeter and reported as a percentage in which normal reading is considered above 95%. The overall extent of pulmonary involvement was determined objectively by the radiologists based on chest computed tomography severity score (CT-SS). The question on having an underlying disease was asked from the participants, but other clinical information was extracted from the patients’ hospital records.

### Measurement of utility value

The third part of the questionnaire included Time Trade-off (TTO) questions to measure the COVID-19 utility value that we used before in a similar study [23]. We first asked the participants to imagine themselves in an unrecovered condition of COVID-19. Then, the respondent was asked how many months (X) of remaining life they would give up to avoid the effects and complications of COVID-19 and live the rest of life in perfect health. In all cases, in the first question, the amount of X was considered as 72 months (6 years). This initial time was selected large enough to avoid framing effect. When the respondent agrees/ disagrees with this baseline point, interviewer increases/ decreases the X number to such an extent that the respondent subjectively becomes indifferent between their current health states in the remaining life-years (Y) and perfect health state in a shorter time and the participant considers equal value for both conditions. Dividing x by y (x/y) defines the COVID-19 disutility, and result of the expression of 1- (x/y) presents its utility for each respondent. To measure the Y amount, we applied the Iranian life table 2016, which defines the gender-age standardized life expectancy [24]. The possible range for the TTO value is between zero for those who are unwilling to lose any time of their life (non-traders) and one for individuals who are willing to lose all their remaining time of life to avoid the disease.

Besides, we used the visual analogue scale (VAS) approach to measure the perceived total current health status. The respondents were asked to rate their HRQoL on a ruler, which was numbered between zero (worst HRQoL) and 10 (best HRQoL).

### Statistical analysis

The descriptive results of the study were presented using statistics of number (with percentage), mean (with standard error), and mean (with interquartile range [IQR]) for all sociodemographic and clinical subgroups. Because the utility variable has right-skewed distribution, which is confirmed by Shapiro-Wilk test, we applied non-parametric tests of Mann-Whitney and Kruskal Wallis respectively to statistically compare the observed differences of utility values for two and more than two groups. The significance level was considered at a *p*-value below 0.05. The expenses were changed from Iran’s national currency to USD using the average exchange rate (USD 1.00 = IRR 160000).

The Two-limit Tobit regression model was applied to determine the effects of sociodemographic and clinical factors on the amount of COVID-19 utility value. Upper and lower limits were set at 1.00 and 0.00 respectively, which corresponds to utility value ranges. In this analysis, we solved the non-normal distribution of the dependent variable, utility value, by taking the logarithm transform. Those variables that had a statistically significant relationship with the level of disease utility in univariate analysis were selected as dependent factors. All statistical analyzes were performed using STATA version 15 (Stata Crop LP, College Station, TX, USA).

## Results

Out of 320 invited treated patients from COVID-19, 287 individuals accepted participation in the study (response rate: 89.69%). Of these, 144 (50.17%) were women, 178 (62.02%) were over 40 years old, and 264 (92.31%) had health insurance. About one-third (96 people) of the respondents were non-traders.

Table 1 presents the descriptive statistics for COVID-19 health utility value by different sociodemographic subgroups. The overall mean (SE) and median (IQR) of the health utility values were 0.863 (0.01) and 0.909 (0.21) respectively. These values for traders were estimated at 0.793 (0.01) and 0.848 (0.17) respectively. The lowest amount of utility value belonged to the elderly (mean (SE) = 0.742 (0.04); median (IQR) = 0.765 (0.42)) and those living in rural areas (mean (SE)) = 0.804 (0.03); median (IQR) = 0.877 (0.30)). Univariate analysis showed that age, place of residence, and household size had a statistically significant effect on disease utility.

**Table 1:** Utility values of COVID-19 disease among different socio-economic subgroups

Socio-Demographic Factors	Number (%)	Utility value		***P***-value
		Mean (SE)	Median (IQR)	
**Total**	287 (100)	0.863 (0.01)	0.909 (0.21)	
Traders	191 (66.55)	0.793 (0.01)	0.848 (0.17)	< 0.01
Non-traders	96 (33.55)	1.00 (0.00)	1.00 (0.00)	
**Age groups**				
Young (< 40 yrs)	109 (37.98)	0.917 (0.01)	0.932 (0.11)	< 0.01
Middle age (40–65 yrs)	115 (40.07)	0.877 (0.01)	0.886 (0.20)	
Elderly (> 65 yrs)	63 (21.95)	0.742 (0.04)	0.765 (0.42)	
**Gender**				
Male	143 (49.83)	0.851 (0.02)	0.907 (0.23)	0.28
Female	144 (50.17)	0.874 (0.01)	0.909 (0.17)	
**Marital Status**				
Single	67 (23.51)	0.889 (0.02)	0.923 (0.08)	0.15
Married	218 (76.49)	0.853 (0.01)	0.899 (0.23)	
**Place of residence**				
Urban	250 (87.11)	0.871 (0.01)	0.920 (0.19)	0.03
Rural	37 (12.89)	0.804 (0.03)	0.877 (0.30)	
**Education**				
Illiterate	70 (25.36)	0.821 (0.02)	0.920 (0.31)	0.06
Non-university	136 (49.28)	0.862 (0.02)	0.899 (0.19)	
University	70 (25.36)	0.893 (0.01)	0.908 (0.15)	
**Employed**				
Yes	125 (44.48)	0.882 (0.01)	0.904 (0.18)	0.09
No	156 (55.52)	0.846 (0.02)	0.928 (0.24)	
**Having basic insurance**				
Yes	264 (92.31)	0.863 (0.01)	0.909 (0.21)	0.76
No	22 (7.69)	0.851 (0.04)	0.889 (0.18)	
**Household dimension**				
< 3 persons	119 (41.46)	0.834 (0.02)	0.889 (0.24)	0.02
> 3 persons	168 (48.54)	0.882 (0.01)	0.924 (0.62)	
**Standardized household’s monthly cost**				
Lowest (< 50 USD)	193 (67.25)	0.870 (0.01)	0.909 (0.19)	0.29
Highest (> 50 USD)	94 (32.75)	0.846 (0.02)	0.906 (0.23)	

Table 2 depicts the findings of estimated utility values of COVID-19 for recovered patients with different clinical characteristics. Out of 287 respondents, 107 (37.28%) had at least one underlying disease, 17 (5.92%) were hospitalized at ICU, 95 (33.10%) were hospitalized for more than 4 days, 217 (65.61%) had SpO2 below normal values, and 33 (11.5%) had moderate and severe pulmonary involvement. Factors of having an underlying disease and disease severity (in terms of hospitalization status and level of lung involvement) had a statistically significant positive effect on obtained utility values. The lowest utility values were recorded for intubated patients (mean (SE) = 0.629 (0.13); median (IQR) = 0.727 (0.33)), for those with severe lung involvement (mean (SE) = 0.651 (0.11); median (IQR) =0.684 (0.24)), and for participants that had underlying diseases (mean (SE) = 0.818 (0.02); median (IQR) = 0.886 (0.26)) respectively. The results also showed that there was no statistically significant difference in currently perceived health status of the studied participants.

**Table 2:** Utility values of COVID-19 disease based on clinical characteristics

Sociodemographic and clinical characteristics	Number (%)	Utility value		***P***-value
		Mean (SE)	Median (IQR)	
**Total**	287 (100)	0.863 (0.01)	0.909 (0.21)	
**Having underlying disease**				
Yes	107 (37.28)	0.818 (0.02)	0.886 (0.26)	< 0.01
No	180 (62.72)	0.889 (0.01)	0.923 (0.08)	
**Patient’s condition**				
Quarantine at home	123 (42.86)	0.896 (0.02)	0.98 (0.13)	0.01
General wards hospitalized	147 (51.22)	0.847 (0.01)	0.886 (0.24)	
ICU hospitalized-non-intubated	13 (4.53)	0.766 (0.06)	0.808 (0.21)	
ICU hospitalized-intubated	4 (1.39)	0.629 (0.13)	0.727 (0.33)	
**Hospitalized days**				
1 day	77 (26.83)	0.872 (0.02)	0.915 (0.19)	0.33
1–4 days	115 (40.07)	0.875 (0.02)	0.931 (0.17)	
Over 4 days	95 (33.10)	0.840 (0.02)	0.896 (0.25)	
**Blood oxygen saturation**				
Normal (over 95%)	70 (24.39)	0.881 (0.01)	0.904 (0.19)	0.33
Below normal (under 95%)	217 (75.61)	0.857 (0.01)	0.923 (0.21)	
**Degree of lung involvement**				
Non/minor involved	64 (22.30)	0.927 (0.01)	0.966 (0.11)	< 0.01
Poorly involved	190 (66.20)	0.844 (0.01)	0.894 (0.24)	
Moderately involved	28 (9.76)	0.859 (0.04)	0.969 (0.20)	
Severely involved	5 (1.74)	0.651 (0.11)	0.684 (0.24)	
**Perceived total health status (VAS**^**a**^**)**				
> 0.7	206 (71.78)	0.869 (0.01)	0.917 (0.17)	0.19
0.5–0.7	23 (8.01)	0.894 (0.03)	0.969 (0.23)	
< 0.5	58 (20.21)	0.826 (0.02)	0.864 (0.26)	

^a^Visual Analogue Scale

Table 3 presents the results of the two-limit Tobit regression analysis. The findings indicated that the participants’ age and hospitalization status were the key determinants of COVID-19 utility value. Therefore, we observed a statistically significant and negative association between age and disease severity with the disease utility.

**Table 3:** Estimation results of the multivariate two-limit Tobit model of utility value for COVID-19 disease

Variables	Coefficient	t statistics	***P***-value	95% CI
**Age**	−0.004	−4.71	< 0.01	[−0.005 – −0.002]
**Place of residence**	0.059	1.46	0.14	[−0.020–0.138]
**Household dimension**	0.013	1.36	0.17	[−0.006–0.032]
**Having underlying disease**	0.016	0.48	0.63	[−0.050–0.083]
**Patient’s condition**	0.107	4.02	< 0.01	[0.054–0.159]
**Degree of lung involvement**	0.036	1.27	0.20	[−0.020–0.092]
**_Cons**	0.485	3.23	< 0.01	[0.190–0.781]

Model statistics; Likelihood ratio = 47.33, *P*-value = < 0.01, Pseudo R2 = 0.302

## Discussion

This multicenter study was performed to estimate the health utility value and its clinical and socio-demographic determinants among newly recovered patients from COVID-19. The overall mean (median) of the disease was obtained as 0.863 (0.909). This means that patients who suffered from COVID-19 lost an average of 13.7% of their HRQoL. However, if we exclude non-traders from the analysis, the mean (median) of the disease utility will decrease significantly by 0.793 (0.848). In other words, people who are severely affected by the disease lose an average of 20.7% of their HRQoL. The findings indicated that 33.35% of the respondents did not accept any time trade-off to get perfect health; meaning that they considered the disease’s effects very insignificant and thought that there was no threat to their health. Further analysis of data confirmed this claim, as 57.29% of non-traders were quarantined at home, compared to 35.60% of traders. Besides, the overall mean of hospitalized days for non-traders and traders was 3.16 and 3.88 respectively.

As expected, there was a significant negative association between the severity of COVID-19 and the disease utility. As the highest mean utility values were observed for those patients who home-quarantined themselves (0.896) and had minor lung infection (0.927), and the lowest mean utility values belonged to the participants who were intubated (0.629) and had severe lung involvement (0.651). In other words, patients who were intubated or had severe lung involvement lost 37.1 and 34.9% of their HRQoL respectively. However, we did not observe such statistically significant associations for SpO2 levels and the number of hospital days. These results suggest that variables of the patient’s hospitalization and lung health status are appropriate indicators for defining the patient’s quality of life and measuring the effects of COVID-19 on their health. However, the two-limit Tobit regression analysis results confirmed only the patient’s hospitalization status as an effective factor on patient’s HRQoL. Depending on the patient’s hospitalization status, a statistically significant difference was seen in the average hospitalization days. This amount was 12.75, 9.31, and 3.19 days for patients admitted to the ICU with intubation and without intubation, and for those hospitalized in the general ward of the hospital, respectively. This result seems logical, because the more severe the disease of COVID-19, the more the patient’s health decreases, and of course the quality of life, in general, and the HRQoL, in particular, reduces.

The findings showed that the severe symptoms of COVID-19 were significantly higher among the elderly than other age groups. As the incidence of underlying disease was 76.19 and 26.34%, the rate of moderate to high pulmonary involvement was 15.88 and 10.27%, and the rate of SpO2 less than normal value was 88.89 and 71.88% among the elderly and other age groups respectively. These observations explained the statistically significant negative association between age and utility values. As age increases, COVID-19 utility value decreases dramatically. This value was 0.917 and 0.714 for the youth and elderly, respectively. This means that COVID-19 damages 8.3 and 28.6% of HRQoL for these two age groups, respectively. Other studies have also confirmed higher morbidity and mortality among elderly patients compared with others [25, 26]. This explanation is also correct for the variable of having underlying diseases. Respondents with underlying diseases showed statistically significant and higher severe symptoms, greater hospitalization rates in the ICU, and severe lung involvement than their counterparts without underlying diseases. Therefore, both age and underlying disease factors can be introduced as confounding variables in our analysis.

These results reflect those of Zhang et al. and Chen et al. who also found that COVID-19 pandemic was associated with mild but significant physical and psychological impairment of Chinese population. Also Chen et al. showed that age and length of stay were strongly associated with the patient’s HRQoL which is in agreement with our findings [20, 27].

In terms of having a detrimental effect on HRQoL of survivors, COVID-19 is comparable with Crohn’s disease, ulcerative colitis [28], thalassemia patients who receive oral iron chelator [29], rheumatoid arthritis [30], and chronic Eustachian Tube dysfunction [31]. Of course, epidemiological indicators, symptoms and effects of these diseases on patients are not comparable. Rather, the patient’s HRQoL is affected similarly from the patient’s perspective. This means that the common point of these diseases is that they destroy a total of 15–17% of patients’ HRQoL. In order to make a logical comparison between these diseases, we must calculate their disease burden, which simultaneously measures and presents both longevity and quality of life lost. In this regard, COVID-19 seems to impose much more burden on society than other diseases, because it has a much higher incidence and mortality rate.

As far as we know, this study, for the first time, calculated and presented the utility value of COVID-19 disease which has various fundamental applications in the burden of disease and economic evaluation studies. To calculate the disease burden, we must separately calculate the indexes of years of life lost due to premature mortality (YLL) and years lived with disability (YLD) [32]. Utility value for the YLL will be zero, but for YLD, we can apply the obtained overall mean (median) value for different health states in this study.

In addition, in cost-effectiveness studies on COVID-19, researchers need to know the health states and associated health utility values. Based on the findings of the univariate and multivariate analysis models, it is recommended that the patient’s hospitalization status (non-hospitalized, general wards hospitalized, ICU hospitalized, and intubated) be used as a reliable proxy to express the severity and grading of COVID-19. Because the symptoms of the disease and its complications have significant variations in different patients, the same criteria cannot be considered for all [33, 34]. Nevertheless, the patient’s hospitalization status can represent all effects of COVID-19. The post-hoc analysis confirmed that the mean (median) of utility values obtained for each of the four conditions had a statistically significant difference compared with its higher-grade at 0.01 significance level.

Multi-centeredness and having a sufficient sample size in different health states were strengths of the current study. However, the findings of the study should be interpreted in light of its limitations. First, we invited the most literate individuals of the family for the interview as a proxy, instead of subjects below 15 years old, which constituted 6.2% (18 individuals) of total participants. This sample selection strategy could lead to over-estimation of the disease utility value because the mean (median) value of the disease utility was 0.966 (0.993) and 0.856 (0.904) for patients younger and older than 15 years, respectively. Second, due to the impossibility of face-to-face interviews with the participants because of the prevention of possible transmission of the disease to the interviewer, the telephone interview may have affected the responses of the participants. Third, while the respondents were newly recovered from the disease, the study’s clinical findings can only be generalized to the survivors. The severity of the disease among recovered participants could be significantly lower than the whole patients.

## Conclusion

Hospitalization status of the patients with COVID-19 is a valid factor in classification of or grading the disease. These patients lost approximately an average of 13% of their HRQoL and the burden of disease caused by COVID-19 appears to be substantial. Patients with underlying disease, hospitalized in the ICU, or those with severe lung involvement have the lowest utility value compared to other patients with the disease, losing about 18–33% of their HRQoL.. Moreover, findings of the regression analysis indicated that the participants’ age and hospitalization status were the key determinants of COVID-19 health utility value.

## Supplementary Information

**Additional file 1.** Questionnaire of eliciting of utility value for COVID-19 Disease (This questionnaire is translated from Persian into the English language).

## Data Availability

The datasets used and/or analyzed during the current study are available from the corresponding author on reasonable request.

## Abbreviations

COVID-19: Coronavirus 2019
SARS-COV-2: Severe acute respiratory syndrome virus coronavirus 2
WHO: World Health Organization
HRQoL: Health-related quality of life
TTO: Time trade-off
RT PCR: Real-time reverse transcriptase polymerase-chain-reaction
ICU: Intensive Care Unit
VAS: Visual analogue scale

## References

[1] Spinelli A, Pellino G (2020). COVID-19 pandemic: perspectives on an unfolding crisis. Br J Surg.

[2] Dashraath P, Jeslyn WJL, Karen LMX, Min LL, Sarah L, Biswas A, Choolani MA, Mattar C, Lin SL (2020). Coronavirus disease 2019 (COVID-19) pandemic and pregnancy. Am J Obstet Gynecol.

[3] Lai C-C, Shih T-P, Ko W-C, Tang H-J, Hsueh P-R (2020). Severe acute respiratory syndrome coronavirus 2 (SARS-CoV-2) and corona virus disease-2019 (COVID-19): the epidemic and the challenges. Int J Antimicrob Agents.

[4] Xu Z, Shi L, Wang Y, Zhang J, Huang L, Zhang C, Liu S, Zhao P, Liu H, Zhu L (2020). Pathological findings of COVID-19 associated with acute respiratory distress syndrome. Lancet Respir Med.

[5] Onder G, Rezza G, Brusaferro S (2020). Case-fatality rate and characteristics of patients dying in relation to COVID-19 in Italy. JAMA.

[6] Li L, Huang T, Wang Y, Wang Z, Liang Y, Huang T, Zhang H, Sun W, Wang Y (2020). COVID-19 patients’ clinical characteristics, discharge rate, and fatality rate of meta-analysis. J Med Virol.

[7] Rajgor DD, Lee MH, Archuleta S, Bagdasarian N, Quek SC (2020). The many estimates of the COVID-19 case fatality rate. Lancet Infect Dis.

[8] Nguyen HC, Nguyen MH, Do BN, Tran CQ, Nguyen TT, Pham KM, Pham LV, Tran KV, Duong TT, Tran TV (2020). People with suspected COVID-19 symptoms were more likely depressed and had lower health-related quality of life: the potential benefit of health literacy. J Clin Med.

[9] Ping W, Zheng J, Niu X, Guo C, Zhang J, Yang H, Shi Y (2020). Evaluation of health-related quality of life using EQ-5D in China during the COVID-19 pandemic. PLoS One.

[10] Rajkumar RP (2020). COVID-19 and mental health: a review of the existing literature. Asian J Psychiatr.

[11] Nair H, Simões EA, Rudan I, Gessner BD, Azziz-Baumgartner E, Zhang JSF, Feikin DR, Mackenzie GA, Moiïsi JC, Roca A (2013). Global and regional burden of hospital admissions for severe acute lower respiratory infections in young children in 2010: a systematic analysis. Lancet.

[12] Worldometer (2020). COVID-19 coronavirus pandemic. Reported cases and deaths by country, territory, or conveyance.

[13] Weissman GE, Crane-Droesch A, Chivers C, Luong T, Hanish A, Levy MZ, Lubken J, Becker M, Draugelis ME, Anesi GL (2020). Locally informed simulation to predict hospital capacity needs during the COVID-19 pandemic. Ann Intern Med.

[14] Moghadas SM, Shoukat A, Fitzpatrick MC, Wells CR, Sah P, Pandey A, Sachs JD, Wang Z, Meyers LA, Singer BH (2020). Projecting hospital utilization during the COVID-19 outbreaks in the United States. Proc Natl Acad Sci.

[15] Carenzo L, Costantini E, Greco M, Barra F, Rendiniello V, Mainetti M, Bui R, Zanella A, Grasselli G, Lagioia M (2020). Hospital surge capacity in a tertiary emergency referral Centre during the COVID-19 outbreak in Italy. Anaesthesia.

[16] Moradi G, Piroozi B, Alinia C, Safari H, Mohamadi P, ZAVAREH FA, Ramezani R, Gouya MM, Nabavi M (2019). Incidence, mortality, and burden of hepatitis B and C and geographical distribution in Iran during 2008–2015. Iran J Public Health.

[17] Alinia C, Mohammadi S-F, Lashay A, Rashidian A (2017). Impact of diabetic retinopathy on health-related quality of life in Iranian diabetics. Iran J Public Health.

[18] Chouaid C, Agulnik J, Goker E, Herder GJ, Lester JF, Vansteenkiste J, Finnern HW, Lungershausen J, Eriksson J, Kim K (2013). Health-related quality of life and utility in patients with advanced non–small-cell lung cancer: a prospective cross-sectional patient survey in a real-world setting. J Thorac Oncol.

[19] van Gestel A, Webers CA, Beckers H, Van Dongen M, Severens J, Hendrikse F, Schouten J (2010). The relationship between visual field loss in glaucoma and health-related quality-of-life. Eye.

[20] Zhang Y, Ma ZF (2020). Impact of the COVID-19 pandemic on mental health and quality of life among local residents in Liaoning province, China: a cross-sectional study. Int J Environ Res Public Health.

[21] Walters SJ (2004). Sample size and power estimation for studies with health related quality of life outcomes: a comparison of four methods using the SF-36. Health Qual Life Outcomes.

[22] World Health Organization. Clinical management of severe acute respiratory infection when novel coronavirus ( 2019-nCoV) infection is suspected: interim guidance, 28 January 2020. World Health Organization. 2020.

[23] Alinia C, Piroozi B, Jahanbin F, Safari H, Mohamadi-Bolbanabad A, Kazemi-Karyani A, Moradi G, Farhadifar F, Ebrahimi M (2020). Estimating utility value for female genital mutilation. BMC Public Health.

[24] WHO (2013). Life tables by country Islamic Republic of Iran.

[25] Spychalski P, Błażyńska-Spychalska A, Kobiela J (2020). Estimating case fatality rates of COVID-19. Lancet Infect Dis.

[26] Baud D, Qi X, Nielsen-Saines K, Musso D, Pomar L, Favre G (2020). Real estimates of mortality following COVID-19 infection. Lancet Infect Dis.

[27] Chen K-Y, Li T, Gong F, Zhang J-S, Li X-K (2020). Predictors of health-related quality of life and influencing factors for COVID-19 patients, a follow-up at one month. Front Psychiatry.

[28] Malinowski KP, Kawalec P (2016). Health utility of patients with Crohn’s disease and ulcerative colitis: a systematic review and meta-analysis. Expert Rev Pharmacoecon Outcomes Res.

[29] Seyedifar M, Dorkoosh FA, Hamidieh AA, Naderi M, Karami H, Karimi M, Fadaiyrayeny M, Musavi M, Safaei S, Ahmadian-Attari MM (2016). Health-related quality of life and health utility values in beta thalassemia major patients receiving different types of iron chelators in Iran. Int J Hematol Oncol Stem Cell Res.

[30] Witney A, Treharne G, Tavakoli M, Lyons A, Vincent K, Scott D, Kitas G (2006). The relationship of medical, demographic and psychosocial factors to direct and indirect health utility instruments in rheumatoid arthritis. Rheumatology.

[31] Teklu M, Kulich M, Micco AG, Brindisi K, Ferski A, Niekamp K, Price CP, Tan BK (2020). Measuring the health utility of chronic eustachian tube dysfunction. Laryngoscope.

[32] Igberaese FI, Iseghohi J (2017). Burden of disease calculation, cost of illness analysis and demand for death: a theoretical review. Int J Dev Manage Rev.

[33] Phua J, Weng L, Ling L, Egi M, Lim C-M, Divatia JV, Shrestha BR, Arabi YM, Ng J, Gomersall CD (2020). Intensive care management of coronavirus disease 2019 (COVID-19): challenges and recommendations. Lancet Respir Med.

[34] Yang P, Wang X (2020). COVID-19: a new challenge for human beings. Cell Mol Immunol.

