# Correction to: The health-related quality of life in Iranian patients with COVID-19

**DOI:** 10.1186/s12879-021-06246-w

**Published:** 2021-06-07

**Authors:** Cyrus Alinia, Safura Yaghmaei, Farman Zahir Abdullah, Asad Ahmadi, Nasrin Samadi, Sima Pourteimour, Hossein Safari, Hassan Mahmoodi, Ghobad Moradi, Bakhtiar Piroozi

**Affiliations:** 1 Department of Health Management and Economics, School of Public Health, Urmia University of Medical Sciences, Urmia, Iran; 2 Nursing Care Research Center, Semnan University of Medical Sciences, Semnan, Iran; 3 Special Education Department, College of Education and Language, Charmo University, Chamchamal, Kurdistan Region Iraq; 4 Social Determinants of Health Research Center, Research Institute for Health Development, Kurdistan University of Medical Sciences, Sanandaj, Iran; 5 Student Research Committee, Urmia University of Medical Sciences, Urmia, Iran; 6 Department of Nursing, School of Nursing and Midwifery, Semnan University of Medical Sciences, Semnan, Iran; 7 Health Promotion Research Center, Iran University of Medical Science, Tehran, Iran

**Correction to: BMC Infect Dis 21, 459 (2021)**

**https://doi.org/10.1186/s12879-021-06170-z**

After publication of the original article [1], an error was identified in two authors’ names.

The incorrect names are:

Sima Pourteymour;

Hassan Mahmoudi

The correct names are:

Sima Pourteimour;

Hassan Mahmoodi

The original article has been corrected.
