# Supplementary material for: The health-related quality of life in Iranian patients with COVID-19
Source: BMC Infect Dis. 2021 May 20;21:459. doi: 10.1186/s12879-021-06170-z (PMC8135385; doi:10.1186/s12879-021-06170-z)
Supplement: Supplementary file 1 — Additional file 1. Questionnaire of eliciting of utility value for COVID-19 Disease (This questionnaire is translated from Persian into the English language). [file 12879_2021_6170_MOESM1_ESM.docx]

**Questionnaire of eliciting of utility value for COVID-19 Disease**

(This questionnaire is translated from Persian into the English language)

| 1. **Patient Demographic Information**   **(This section is completed by interviewing the patient and using the patient's medical record)** |
| --- |
| 1. Patient code: ------ |
| 1. What is your province of residence?   Kurdistan □ West Azerbaijan □ Hamadan □ |
| 1. In which of the following areas have you lived during the past year?   Urban □ Rural□ |
| 1. Patient gender:   Male □ Female□ |
| 1. Patient age:   ----- Year & ----- Month |
| 1. What is your marital status?   Single □ Married □ |
| 1. What is your level of education?   Illiterate □ Non-university □ University □ |
| 1. Do you have basic health insurance?   Yes □ No □ |
| 1. Do you have a job?   Yes □ No □ |
| 1. How many people do you live under the same roof?   ------ person/s |
| 1. What has been your total household expenditure over the past month?   ------ Rial |
| **B) Patient Clinical Information**  **(This section is completed by using the patient's medical record)** |
| 1. Does the patient have an underlying disease?   Yes□ No□ |
| 1. If yes, please mention the name of the disease/s.   -------- |
| 1. Determine the patient's hospitalization status.   Non-hospitalized □ General wards hospitalized □  ICU hospitalized-no-intubated □ ICU hospitalized-intubated□ |
| 1. What was the number of days the patient was hospitalized?   ------ |
| 1. What was the minimum blood hemoglobin oxygen saturation (SpO2) of the patient during the hospitalization period?   ------ |
| 1. What is the level of lung involvement of the patient based on the results of CT-scan and diagnosis of the relevant radiologist?   None/ minor □ Poor □ Moderate □ Severe □ |
| **C) Estimation of Health-Related Quality of Life**  **(This section is completed by interviewing the patient)** |
| 1. If zero indicates death and 100 indicates complete health, how healthy do you think you are? …… |
| 1. To answer the following questions, please imagine yourselves in an untreated condition of COVID-19 disease.   Suppose a free treatment is developed that cures your Covid-19 disease and returns you to normal and perfect health. This treatment method has no side effects, is entirely safe, and will definitely cure you immediately, but instead, it will shorten your life by six years.  Are you willing to accept this treatment? Yes □ No □  **Interviewer guide:**  [If the patient's answer is "yes", increase the number of traded years annually and ask the question again. This increase continues until the interviewee no longer accepts more than that]  [If the patient's answer is "no", decrease the number of traded years annually and ask the question again. This decrease continues until the interviewee no longer accepts less than that]  What the maximum number of years the patient is willing to lose to receive this treatment:  ……..  (For example: 5 years, 6 years and 3 months, 7 years and 8 months ...) |
